# Editorial: Novel and emerging therapies in heart failure

**DOI:** 10.3389/fcvm.2023.1179352

**Published:** 2023-03-23

**Authors:** Grigorios Giamouzis, Andrew Xanthopoulos, Konstantinos Dean Boudoulas, Georgios Karagiannis, John Skoularigis, Filippos Triposkiadis

**Affiliations:** ^1^ Cardiology Department, General University Hospital of Larissa, Larissa, Greece; ^2^ Faculty of Medicine, School of Health Sciences, University of Thessaly, Larissa, Greece; ^3^ Division of Cardiovascular Medicine, The Ohio State University, Columbus, OH, United States; ^4^ Transplant Department, Harefield Hospital and Cardiology Department, Hillingdon Hospital, London, United Kingdom

**Keywords:** heart failure, acute, chronic, novel therapies, emerging therapies, comorbidities, HFrEF – heart failure with reduced ejection fraction, HFpEF – heart failure with preserved ejection fraction

**Editorial on the Research Topic** Novel and emerging therapies in heart failure

Heart failure (HF) has been named “the growing epidemic” (1, 2). Over the last decade, the annual number of HF hospitalizations has almost doubled with approximately 50% of patients being rehospitalized within 6 months of discharge (3). The complex array of physiologic, psychological, social, and health care delivery issues makes it a challenging chronic disease to manage. Understanding the epidemiology and pathophysiology of the syndrome, identifying the predictors and their strength of association with outcomes, and using the available diagnostic modalities cost-effectively are essential in order to implement novel therapeutic approaches to curb this epidemic.

This research topic was initiated by Triposkiadis et al. and published in 2022 in Frontiers in Cardiovascular Medicine. Our aim was to invite articles highlighting current research in novel or emerging therapeutic approaches -pharmacological and invasive- in both the acute and chronic setting of the disease, as well as potential interaction and/or barriers with concomitant cardiovascular or non-cardiovascular diseases. In this special issue, we present 7 original research articles, 3 systematic review articles and 1 opinion article that address such issues and investigate why, despite the emergence of novel therapeutic approaches that promise life prolongation and hospital length reduction, this patient population often has a poor prognosis and often requires rehospitalization. Below is a summary of these articles.

An increased number of HF patients suffer from chronic angina, which seriously impairs their quality of life (4). Cardiac shock wave therapy (CSWT) has recently become appealing due to the improvement in angina symptoms. In a systematic review and meta-analysis by Quan Qiu et al. the efficacy of CSWT on coronary artery disease (CAD) patients was evaluated. A total of 8 randomized controlled trials (RCTs) and 2 prospective cohort studies involving 643 patients (*n* = 336 CSWT and *n* = 307 control) were included in the study. CSWT was shown to moderately improve myocardial perfusion and cardiac function among patients with CAD, a finding that may provide the clinicians with a meaningful and valuable option for revascularisation, for instance in selected HF patients with ischemic cardiomyopathy.

Puerarin (7,40-dihydroxy-8-C-glucosylisoflavone) is the major bioactive ingredient of the root of Radix Puerariae and has been widely applied for the adjunctive management of CAD; its main drug delivery method is intravenous injection (5). Zunjiang Li et al. conducted a systematic review and meta-analysis that aimed to assess the adjunctive efficacy and safety of Puerarin injection (PI) in acute HF patients. Eight studies were included with a total of 614 patients with acute HF that demonstrated that adjunctive treatment with PI was superior to, and as safe as, conventional medicine alone. Specifically, PI increased the total effective rate and improved left ventricular ejection fraction (LVEF) as compared with conventional therapy without raising safety concerns, as there was no significant difference in adverse events between the two groups.

The pharmacological management of heart failure with preserved ejection fraction (HFpEF) continues to be an area of uncertainty due to multiple studies failing to show a clear benefit associated with therapies that have otherwise proven useful in the management of other HF subtypes. Therefore, both the 2021 ESC and the 2022 AHA/ACC/HFSA updated HF guidelines state that “in the absence of recommendations regarding disease-modifying therapies, treatment should be aimed at reducing symptoms of congestion with diuretics” (6, 7). Based on the results of 10 current RCTs comprising 10,334 patients, Danning Yang et al. performed a meta-analysis to illustrate the therapeutic impact of sodium-glucose cotransporter-2 (SGLT2) inhibitors in HFpEF patients. Treating HFpEF patients with SGLT-2 inhibitors was associated with a 22% reduction in the composite outcome of cardiovascular death or hospitalization for HF and had a similar improvement in health-related quality of life; interestingly, no statistical difference was observed in 6MWT distance. In an opinion article, Luis Tolento Cortes and Lisa Hong highlight the flaws of all recent RCTs regarding the use of ACEIs/ARBs/ARNIs, beta-blockers, aldosterone antagonists and SGLT2 inhibitors in patients with HFpEF. In a subsequent original research article, María Valero-Muñoz et al. identified the cardiac-specific features of protein and phosphoprotein changes in a murine model of HFpEF using mass spectrometry. Proteomics analysis of the left ventricular (LV) tissue showed that almost 900 proteins were expressed differentially between HFpEF and sham mice including changes in sarcomeric proteins, mitochondrial-related proteins, and NAD-dependent protein deacetylase sirtuin-3 (SIRT3). In summary, this study demonstrates marked changes in proteins related to mitochondrial metabolism and the cardiac contractile apparatus in HFpEF; the authors propose that SIRT3 may play a role in perpetuating these changes and, therefore, may be a target for drug development in HFpEF.

Yishu Wang et al. examined the effects of early phase 1 cardiac rehabilitation (CR) on cardiac function in patients with coronary heart disease (CHD) and acute HF using impedance cardiography (ICG). A total of 98 patients were recruited and randomized into two groups. The control group received standard pharmacotherapy and the CR group received standard pharmacotherapy combined phase 1 CR. NT-proBNP and hemodynamic parameters measured by ICG were estimated at baseline and at the end of the treatment period. Phase 1 CR resulted in a more pronounced reduction in NT-proBNP levels. Similarly, most hemodynamic parameters improved in the CR group, but not in the control group. These findings suggest that phase 1 CR in combination with the standard pharmacotherapy could improve hemodynamic characteristics by elevating cardiac output, ameliorating preload, improving systolic and diastolic function, and relieving afterload. Therefore, suitable stabilized patients with CHD and acute HF should undergo phase 1 CR.

In a phase 1, open-label, single-arm, first-in-human study, Lien-Cheng Hsiao et al. aimed to assess the safety and efficacy of combined intracoronary (IC) and intravenous (IV) transplantation of umbilical cord-derived mesenchymal stem cells (UMSC01) for heart repair in patient with a ST-elevation myocardial infarction (STEMI) with impaired LVEF (30%–49%) following successful reperfusion by percutaneous coronary intervention. In the 6 subjects who completed the study, there were no treatment-related serious adverse events or major adverse cardiovascular events during infusion or follow-up. NT-proBNP levels and wall motion scores decreased, whereas the LVEF increased significantly at the 12-month follow-up compared to the baseline values. This pilot study showed that combined IC and IV transplantation in this patient population appears to be safe, feasible, and potentially beneficial in improving heart function. Phase 2 studies will be needed to further investigate the effectiveness of dual-route transplantation of UMSC01 in STEMI patients.

In another interesting original research study by Bin He et al. the pathogenesis, immune-related pathways and important biomarkers involved in the progression of cardiomyopathy due to various etiologies were investigated. The authors reported that the hub genes (CD14, CCL2, and SERPINA3) can be used as markers to distinguish patients with cardiomyopathy. Furthermore, the authors demonstrated that the innate immune response, either dysregulation/imbalance of innate immune cells or activation of adaptive immune responses, were involved in the progression of HF.

Red blood cell distribution width (RDW) is a simple hematological parameter that reflects the heterogeneity of the red blood cell size (anisocytosis); higher levels of RDW have been associated with poor outcome in patients with HF (8, 9). Recent studies suggest that the favorable pivotal mechanism of SGLT-2 inhibitor in patients with HF is the stimulation of erythropoiesis *via* an early increase in erythropoietin (EPO) production, which leads to a rise in hematocrit (10). However, EPO has been also implicated in the pathophysiology of RDW increase in this patient population. In a very interesting mechanistic study by Nikolaos Katsiadas et al. the effects of SGLT-2 inhibitor administration on RDW in patients with HF and diabetes mellitus (DM) were examined. RDW increased with time in patients who received dapagliflozin and this increase was mainly attributed to the induction of hemopoiesis from dapagliflozin. Interestingly, increased baseline, but not post-SGLT2 inhibitor therapy, RDW values were independently associated with poor outcomes in patients with HF and DM.

Left bundle branch pacing (LBBP) is emerging as an effective alternative to achieve cardiac resynchronization therapy (CRT) and improve heart function (11). In a retrospective study by Ying Gu et al. the feasibility and efficacy of LBBP in HF patients with a LVEF <50% and left bundle branch block (LBBB) was investigated; the study suggested that LBBP using a low and stable pacing threshold was feasible with a high successful implantation rate and was effective in correcting LBBB and improving LV structure and function.

Finally, Zhang Fang et al. aimed to evaluate the proper energy intake patterns and daily calorie intake in adult patients with HF in the United States of America. Among almost 1,000 participants, moderate malnutrition was more frequently related with mortality. Low-carbohydrate pattern (LCP) and median-carbohydrate pattern (MCP) diets had a lower risk of death as compared to a high-carbohydrate pattern (HCP) diet. There was no association between different amounts of calorie intake and all-cause mortality. Therefore, the relationship between energy intake and all-cause mortality may be influenced by energy intake patterns in HF patients.

Research in HF is an exciting and continuous endeavour. The hope is that this special edition on *Novel and Emerging Therapies in Heart Failure* will provide a meaningful contribution to the literature, will shed light to the understanding of HF, and will open new treatment pathways for the benefit of our patients.
